# Microbiota: Overview and Implication in Immunotherapy-Based Cancer Treatments

**DOI:** 10.3390/ijms20112699

**Published:** 2019-05-31

**Authors:** Giovanni Brandi, Giorgio Frega

**Affiliations:** Department of Experimental, Diagnostic and Specialty Medicine, Sant’Orsola-Malpighi Hospital, University of Bologna, 40138 Bologna, Italy

**Keywords:** microbiota, microbiome, immunotherapy, adoptive cell transfer (ACT), CAR T-cell, TCR, TIL, checkpoint inhibitors, immuno-oncology, cancer, diet

## Abstract

During the last few years, the gut microbiota has gained increasing attention as a consequence of its emerging role as a modulator of the immune system. With the advent of the era of checkpoint inhibitors immunotherapy and adoptive cell transfer (ACT) in oncology, these findings became of primary relevance in light of experimental data that suggested the microbiota involvement as a plausible predictor of a good or poor response. These remarks justify the efforts to pinpoint the specific actions of the microbiota and to identify new strategies to favorably edit its composition.

## 1. Introduction

In the last two decades, intestinal microbiota, a silent and forgotten, but capital player of health, has finally been recognized in its own role concerning human physiology and pathology.

Initially hypothesized to be limited to the gastrointestinal tract, its role is now suggested to be much larger, including immune-modulatory effects outside the gut and even impacting on several brain functions.

Meanwhile, we experienced the dawn of immunotherapy in the treatment of hematological and solid tumors. The immunotherapies already approved, and the new concept ones, such as cutting-edge types of adoptive cell transfer (ACT) therapy, are promising to gain an ever-increasing relevance within the landscape of cancer treatments.

Here we summarize some general aspects of human microbiome, focusing on specific immunomodulatory functions and on its emerging role as modulator of response to cancer immunotherapies.

## 2. The Human Microbiota: Overview

Only recently the concept of humans as not merely autonomous eukaryotic organisms, but rather as ‘holobiots’ (the host plus his connected microbial network) reached the spotlights [1,2].

All in all the human microbiota has been estimated to contain near to 1 × 10^14^ colonizing bacteria, over one hundred and sixty bacterial species in each individual (of more than one thousand identified), and millions of genes [3,4,5,6].

This huge bacterial population may reside within and colonize the gastrointestinal tract (i.e., autochthonous bacteria) or pass transiently through the gastrointestinal tract (i.e., allochthonous bacteria). Autochthonous bacteria should be considered dominant (>10^7^ CFU/g) or subdominant (<10^7^ CFU/g) depending on their concentration [7,8]. This is significant because the effect on the host relies on the amount of producing bacteria, especially if mediated by bacterial metabolites [8].

In the large bowel, the anaerobic–aerobic ratio varies, being lower on the mucosal surface and higher in the lumen [9]. The intestine in newborns is sterile, but bacterial colonization quickly occurs with pioneer facultative anaerobes bacteria, coming from the environment and the mother. These microbes burn out the oxygen in the colonic lumen, thereby creating propitious environmental conditions for the spread of strict anaerobes. Actually, the anaerobes will then become the vast majority (dominant population), while the other bacteria will be only metabolically minor players; however, their role in immune regulation cannot be excluded [10,11].

The early colonization and composition of the gut microbiota play a relevant role in shaping the immune system and have delayed consequences, affecting the risk of developing several diseases such as asthma, allergies, and inflammatory bowel disease (IBD) [12]. The real impact of these early-in-life events on risk and treatment of neoplastic diseases has not been completely clarified yet.

The adult human gut tract, as mentioned, hosts an extremely complex and dynamic microbial ecosystem playing a crucial role in the regulation of both enteric and systemic homeostasis. Its composition has been studied by traditional cultural methods for centuries. However, traditional bacterial culture methods permit the culture of a limited portion (<50%) of bacteria [13,14].

Recently, molecular techniques with 16S rRNA or DNA/sequencing/metagenomics approaches provided greater information about both taxonomy and the whole genome of microbiota (so-called microbiome), unraveling several potential functions of gut microbes. The 16S rRNA technique relies on the isolation and sequencing of the 16S rRNA gene, which encodes for the 16 rRNA, the structural component of the small ribosomal subunit. The 16S rRNA gene contains hypervariable regions which lead to a sequence peculiarity among bacterial species [15]. Metagenomics analysis relies on the study of the nucleic acids of a community of organisms extracted from the environment [16]. Metagenomics approaches can be “targeted” to the analysis of a specific region (such as the 16S rRNA gene sequence) or “untargeted” (or “shotgun”), namely on the basis of the sequencing of all microbial genetic material contained in the specimen [17,18]. Unfortunately, these non-culture-based approaches also suffered for several limitations, mainly linked to their specific methods. Furthermore, the molecular approaches do not allow bacterial strains for in vivo experiments using gnotobiotic animal models. In summary, on the one hand, less than 20% of bacteria grown from stool are detectable with metagenomics [19]; on the other hand, a large number of bacteria detected in feces are nonviable. In this context, improved culture methods are still an absolute necessity.

More recently, culturomics approaches that couple cultivation of living bacteria using several culture media with MALDI-TOF for rapid identification of the strain, increase the number of species detectable in the human gut [20]. The definition of taxonomic hierarchy by the operational taxonomic unit (OTU) shows that microbiota is organized along several levels of similarity (from phyla to strains), going from >99% of sequence similarity for bacterial strains to <90% of similarity for phyla levels.

Only limited types of bacteria can colonize the gut. The majority of human bacteria belong to at least four phyla: *Firmicutes*, *Bacteroidetes*, *Actinobacteria*, and *Proteobacteria* [21,22], and to six genera of strict anaerobes: *Bacteroides*, *Eubacteria*, *Bifidobacteria*, *Clostridia*, *Peptostreptococci*, and *Ruminococci*. *Firmicutes* and *Bacteroidetes* are the dominant phyla [22].

Notwithstanding a unique and distinct microbial pattern that every subject has, like an adjunctive fingerprint, intestinal microbiota seems not built in a random fashion, but stratified along main clusters (enterotypes) based on *Bacteroides*, *Prevotella*, and *Ruminococcus* genera. Subdominant bacteria support metabolic profiles of enterotypes, because defined functions are shared among different bacteria indifferently, by their numerousness [23].

A further key point concerns the relationship of the human gut microbiota and the gastrointestinal tract, in terms of both its anatomical distribution and relationships with the mucosa. These aspects are very different in humans and in rodents, and this suggests caution in translating data generated in rodents to human beings [24].

Actually, the bacterial density in the human small bowel is relatively low, increasing from the duodenum (≃10^1–3^ CFU/mL) to the ileocecal valve (≃10^10^ CFU/mL) and reaching the highest concentration in the colon (≃10^11–12^ CFU/mL) [25,26,27]. Conversely, in rodents, the number of endoluminal bacteria along the whole alimentary tract is less variable. Even the relationship between the microbiota and the intestinal epithelium is different between rodents and humans. First of all, the anatomy of the intestinal tract is significantly dissimilar between the two species. There is a discrepancy in terms of the relative extent of the digestive tract (in relation to the whole body size) [24]. Furthermore, even if the ratio between the entire intestinal surface and the whole body surface is similar [28], it is not the same when focusing on distinct tracts of the gut [29]. The small intestine:colon length ratio and the small intestine:colon surface ratio are more than two times and more than twenty times higher in humans than in mice, respectively [28,29,30]. There are also great differences in terms of length of the intestinal villi and anatomical structure of the intestinal wall [29]. As in humans, two distinct layers of mucus line the mouse colon epithelium [31]. Much less is known about the bacterial–epithelium interaction in the murine small intestine [32]. Undoubtedly, the epithelial RegIIIγ secretion plays a cardinal role in preserving a spatial separation (approximately 50 μm) between the epithelium and the microbes, as shown by pieces of evidence in Myd88−/−mice [33]. Nevertheless, focusing on this research, it is important to bear in mind that also in wild-type mice the mucosa-associated microbes are not completely absent, even if they are in a significantly lower amount when compared with cohoused Myd88−/− littermates [33].

In rodents, there is probably an intimate relationship between the intestinal mucosa and a large number of bacteria, often found to cluster over the mucus gel or in direct contact with epithelial cells. In humans, such great proximity is lacking.

**Figure 1:**
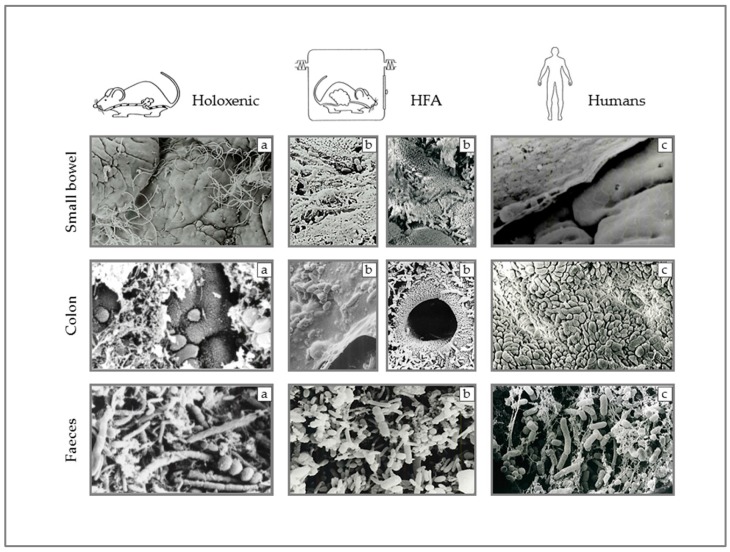
Scanning electron microscopy images of small bowel mucosa, colon mucosa, and fecal bacteria in holoxenic (i.e., raised under conventional circumstances) mice (**a**), HFA (human-flora-associated mice) mice, namely germ-free mice inoculated with components of the human flora (**b**), and humans (**c**).

In particular, human colonic epithelium beneath the mucus layer remains overwhelmingly germ-free under normal conditions [34]. We described this aspect using a scanning electron microscope, afterwards confirmed by different techniques, nearly twenty years ago [35] (Figure 1).

Intraluminal bacteria are stratified through the existence of a mucous layer and the activity of immunoglobulins (IgA) yielded by plasma cells in the lamina propria and transferred within the gut lumen by transcytoses [36].

The mucus occurs in two distinct physical forms: a thin layer of stable, insoluble mucus gel firmly adhering to the intestinal mucosal surface and a soluble mucus, quite viscous, but that mixes with the luminal juice and plays a crucial role in regulating the relationships between bacteria and the colonic mucosa [31]. The inner stable mucus is impervious for bacteria that, conversely, can be found in the outer loose mucus layer [37]. This latter mucus is continuously secreted and then shed, discarded, or digested by specific bacteria [38].

Moreover, the thickness of mucus in humans (50–450 mm) is approximately double that in rodents. It is the mucus layer, together with the innate immune system that, at least in mice, actively contains microbiota, mainly in the lumen, limiting penetration into the mucosa and avoiding excessive pro-inflammatory signaling [39]. FISH analysis of colon biopsies of healthy subjects confirmed that the number of bacteria on the mucosa is also lower (<10^7^ CFU) than in feces and large zones of the mucus layer are often free from bacteria [40,41,42,43].

Clearly, it will be impossible and counterproductive (as showed by germ-free animal experiments) to obtain persistent and complete isolation along of the entire size of the intestinal surface. Physiologically, commensals can induce the secretion of mucin and antibacterial peptide (such as defensins) by epithelial cells, the recruitment of immune cells to the mucosa, and the maturation of GALT (Gut-Associated Lymphoid Tissue) [25,44]. These microbes can also sometimes reach the lamina propria, where they are sampled and removed by means of macrophages or dendritic-cells-mediated phagocytosis.

Bacteria can persist alive within dendritic cells and induce a mucosal IgA immune response [45]. Live-carried bacteria can induce a stronger IgA plasma cells response than killed ones [25]. Loaded dendritic cells are then confined by mesenteric lymph nodes and cannot roll in the other systemic secondary lymphoid tissues [25,45].

Mucus likely plays an indirect role also in microbiota-related GALT genesis and even in immunity response at distance from the GI tract. The immune system is organized at various levels (molecular, cellular, and systemic) in order to discriminate among a range of stimuli [46], some of which are able to provoke or activate a response leading to immunity (for pathogens, neoplastic, and grafted cells) and inhibit some others, leading to tolerance for both normal microbiota and dietary antigens.

## 3. Microbiota: Physiological Fluctuations and Induced Disruptions

The microbiota has different characteristics during life, and these changes, in physiological conditions, are mainly driven by diet changes. During childhood, *Bifidobacteria* initially dominate the microbiota [47,48], countering the pro-inflammatory environment typical of the gastrointestinal (GI) tract at this stage of life. In adults, the microbiota is mainly represented by *Firmicutes* and *Bacteroides*, able to provide SCFA (short-chain fatty acids) to the host, digesting plant polysaccharides (otherwise indigestible), thus increasing the ability to extract energy from the diet [49].

In old age, there is a progressive loss of bacterial biodiversity, with an increase in pathobionts (as *Fusobacteria*) and a rearrangement of bacteria producing butyrate (*F. prausnitzii*/*Roseburia* vs. *Eubacterium limosum*) [50]. In centenarians, bacterial clusters are selected that potentially may interfere with the immune response (*Akkermansia* and *Christensenellaceae*) [51,52].

Although these physiological changes in microbiota composition during life are related to diet changes, recently it has been suggested that other factors may be involved: the geographic origin of the subject and ethnicity [53,54].

Finally, additional conditions may induce dysbiosis, such as the use of antibiotics. The latter deeply impacts the bacterial ecology of the gut. A five-day treatment with broad-spectrum antibiotics, administered to healthy subjects, may induce depletion of some bacterial strains (*Bifidobacteria*) and an explosion of pathobionts (*E. faecalis* and *F. nucleatum*) [55]. The same authors also reported that more than a month is required to restore a near-previous composition and a few common species remain undetectable longer [55]. These disruptions can probably strongly interfere with the systemic immune response [56].

## 4. Digest on Immuno-Oncology Landscape

Immunoescape is one of the hallmarks of cancers [57]. Cancer cells are able to generate an immunosuppressive microenvironment that allows them to grow and to avoid immune destruction. Nevertheless, the immune system does not have a passive role in tumor evolution. The immunoediting hypothesis (elimination, equilibrium, escape) confers to the immunity the ability to sculpt the immunologic phenotype of the tumor [58]. According to this, cancers acquire an immuno-imprinted habitus that confers them an evolving ability to suppress or to escape from the immune system [58].

Since the first FDA (Food and Drug Administration) approval of ipilimumab in melanoma patients in 2011, checkpoint inhibitors revolutionized the landscape of cancer treatments. These treatments basically target proteins that physiologically suppress the immune system, avoiding abnormal immune responses. In addition to the already approved anti-CTL4-mAb and anti-PD-1/PD-L1-mAbs, other innovative agents aimed at activating the antitumor T-cell response or targeting other inhibitory receptors (e.g., Tim-3, VISTA or Lag-3) are currently under investigation in solid tumors [59,60].

Unfortunately, despite the exciting, durable response sometimes obtainable, not all patients and not all malignancies are susceptible to immunotherapy to date.

The reasons for the lack of response of some tumors are not completely understood, even if probably it mostly depends on defects in antigenicity and adjuvanticity, which are keys factor in shaping the immunogenicity of tumor cells [61]. To date, several biomarkers (PD-L1 expression, tumor-infiltrating lymphocytes, mutational burden, immune gene signatures, etc.) have been proposed, even if they are not always predictive alone due to lack of sensibility or sensitivity [62]. The level of somatic mutations seems to be a crucial factor. Tumors with a high number of somatic mutations (i.e., melanomas and smoking lung cancers) are more responsive than low rate ones (i.e., gastrointestinal cancers and breast) [63].

Adoptive cell therapy (ACT) is a new and promising strategy to immunologically fight cancer. Only a few months ago, the FDA approved autologous T cells (elaborated to express a chimeric anti CD-19 B lymphocyte antigen) for the treatment of diffuse large B-cell lymphoma and acute lymphoblastic leukemia (children and young adults) in relapsed or refractory setting [64].

**Figure 2:**
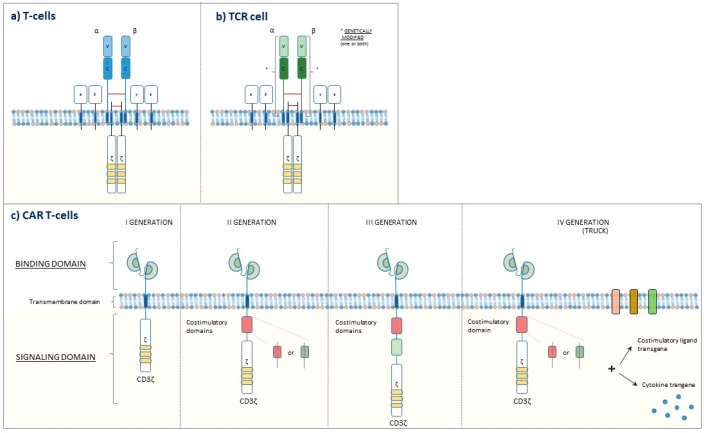
(**a**) T-cell receptor on the surface of T cell, (**b**) engineered T-cell receptor on the surface of engineered T cell (TCR cell), (**c**) Four generations of chimeric antigen receptor (CAR) T cells. I generation: the intracellular signaling domain alone (CD3 ζ-chain). II generation: one costimulatory domain and the intracellular signaling domain. III generation: two costimulatory domains and the intracellular signaling domain. IV generation: costimulatory domain(s), the intracellular signaling domain, and activity enhancing factors (e.g., cytokines, co-stimulatory ligands).

This groundbreaking weapon lies in the patient’s leukapheresis, T-cell engineering on the bench to express a chimeric antigen receptor (CAR) specific against a defined tumoral antigen, and finally reinfusion, usually after preconditioning lymphodepletion. A similar strategy consists of reinfusion of T-cell receptor (TCR)-engineered T cells, which possess a genetically modified receptor brought against tumoral antigens and comparable to a natural T-cell receptor (Figure 2).

The availability of ever-honed gene-editing technologies promises to lead to further evolutions, such as the development of allogeneic T cells generated by healthy donors [65]. Furthermore, newly developed CAR T cells such as tandem CAR T cell (which harbor two ligand-binding domains), multi-CAR T cells (which harbor different chimeric antigen receptors), built-in-CAR T cells (which are modified to release anti-PD-L1 antibodies within the tumor), and many others have already been generated, mainly with the aim to potentiate the efficacy or reduce the toxicities [66,67,68].

Unfortunately, this approach seems not to be equally effective in hematological and nonhematological malignancies, mainly due to the absence of properly tumoral-specific antigens and to surface cellular antigens heterogeneity [69]. Moreover, even achieving a potent antitumoral efficacy, there remains the issue of serious adverse events, notably in brain tumors [70]. Despite that, the advances in the knowledge of cancers immunogenetics, tumoral antigens, and the advent of new technologies to curtail side effects will favor the advent of these therapeutic approaches.

Further cell therapies, based on the reinfusion of autologous tumoral-infiltrating lymphocytes (TIL) or autologous/engineered natural killer cells (NK) expanded in vitro after patient’s systemic lymphodepletion, showed encouraging results in some cancer types and appear more promising in the foreseeable future, even if the proper sequence and the real gain with respect to other disposable immunotherapies will have to be defined [71,72].

Adoptive therapy with tumoral-infiltrating lymphocytes, screened for their activity against mutant cancer proteins, and then amplified, seems to be able to achieve striking responses in selected patients, even if these therapies are at the dawn [73,74].

Furthermore, the considerable researches that foster these approaches are allowing unique somatic mutations (specific of each singular malignancy and hardly ever shared between and also within distinct cancer types) to be identified that can lead an anti-tumoral response [75].

Finally, along the lines of what happens with checkpoint inhibitors, not all subjects respond well to these futuristic treatments to date and some patients explore relevant toxicities [76,77].

## 5. Microbiota: Implications in Immuno-Oncology

As mentioned above, the intestinal immune system probably has the heaviest and the most fragile task within the entire host immune system, facing a huge amount of alimentary and microbial antigens during the entire lifetime.

The vast majority of the current knowledge on microbiota as an immune system modulator originates from germ-free animal model studies. These animals are raised under sterile conditions and the following exposure to single or small microbial communities (gnotobiotic animals) allow the investigations on the interactions between each species and the host [36].

Data obtained by this type of research showed how some bacteria (*Clostridium* cluster 4 and 14) may enhance the anti-inflammatory branches of the adaptive immune system, inducing a peripheral expansion of Foxp3+ Tregs [78,79]. These regulatory T cells (Tregs) are able to produce IL-10 (and other molecules such as CTLA-4, IL-2, IL-10, TGF-β, IL-35, and more) thus leading to immune-tolerance and immunosuppression [80,81]. In light of this, these lymphocytes play a key role in maintaining the immunological self-tolerance, preventing autoimmunity [81]. By way of example, Tregs-derived IL-10 cytokine exerts a key role in safeguarding the right immune balance at the sites of environmental exposed surfaces (i.e., lung and gut) [80]. The role of Tregs in cancer is likewise crucial. Several preclinical pieces of evidence showed how these cells are able to hamper the immune response against cancer [82,83]. Consequently, Tregs are considered an attractive target for cancer immunotherapies [81]. Furthermore, a recent study suggested that PD-1+ regulatory T cells could be responsible for hyper-progression to anti–PD-1 immunotherapy [84].

**Figure 3:**
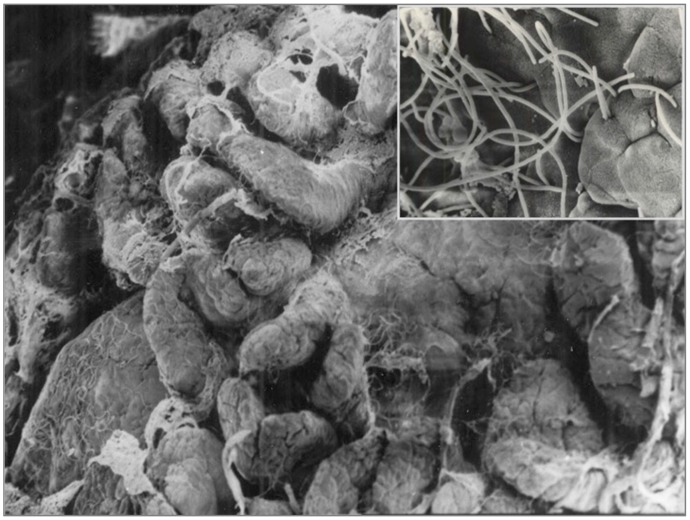
Scanning electron microscopy images, showing SFB (segmented filamentous bacteria) inside and outside the Peyer’s patches.

Conversely, the pro-inflammatory component of GALT is induced in rodents from SFB that alone are able to replace the whole activity of microbiota for this specific characteristic [85]. These bacteria have the ability to penetrate the epithelium of both the small intestine and cecum of rodents, not only on the Peyer’s patches, but also elsewhere (Figure 3).

In this way, they lead to IL-17, IL-23, and IL-6 release by dendritic cells, as well as to T-helper 17 recruitment. These bacteria play a pivotal role in the GALT formation in rodents, however, they have never been detected in adult human microbiota [86]. Conversely, some recent reports suggest their existence and their potential role at an early age [87].

Furthermore, the belief that organs and tumors are absolutely sterile sanctuaries has recently collapsed [88]. Some bacterial species have been established to be able to accompany the neoplastic growth of colon–rectal cancers and to migrate in metastatic sites [89]. Moreover, recent data showed that an unexpected presence of bacteria within tumor tissue, even in malignancies beyond the gastrointestinal tract, could modulate the immune response by inducing immune suppression. For instance, the endogenous microbial population in pancreatic ductal adenocarcinoma, which is more abundant than in a normal pancreas, suppresses monocytic differentiation, so inducing T-cell anergy [90]. Similarly, *Fusobacterium nucleatum* has been detected in certain colon cancers, both in primitive tumors and in liver metastases, and has been reported to correlate with a worse prognosis [91]. Furthermore, antibiotic treatments delay the tumor growth of patients’ xenograft mice-derived from *F. nucleatum*-positive colon rectal tumors [89]. It has also been described that *F. nucleatum* can interact with receptors of the innate immune system (TLR4) by modulating autophagy, by decreasing apoptosis and by inducing chemoresistance [92].

The role of intratumoral bacteria as a potential reason for chemoresistance could also result from bacterial metabolic functions, as reported for gemcitabine in a colon cancer mouse model [93]. A previous study showed the ability of certain bacteria to influence (to impair but also to improve) the efficacy of chemotherapeutic agents in vitro [94]. The same authors validated the negative impact of intratumoral bacteria on the efficacy of a sample drug (i.e., gemcitabine) in vivo [94]. These findings are credibly expected to outbreak new frontiers in the fields of cancer-immune-escape mechanisms and drug resistance.

Moving on to the anti-cancer immune response, about ten years ago a group of scientists described how microbiota or, more precisely, some subdominant bacterial species can induce the recruitment of T cells within organs in mice [95]. Other studies revealed the importance of microbiota in modulating the efficacy of certain chemotherapies (i.e., oxaliplatin, cyclophosphamide) by promoting an immune response against the tumor [96,97].

Along these lines, Sivan A. and coauthors formulated the hypothesis and elegantly demonstrated how microbiota could also play a major role in shaping the anticancer immune response and tumor growth. Genetically identical mice imported from two different facilities (and consequently with different microbiota composition) displayed a dissimilar response to immunotherapy. Conversely, no differences were reported by cohousing mice. Furthermore, direct administration of *Bifidobacterium spp.* improves tumor-specific immunity and response to anti-PD-L1 immunotherapy by activating intratumoral antigen-presenting cells [98]. An analogous research revealed the lack of response to CTLA-4 blockade in antibiotic-treated or germ-free mice and allowed the identification of bacterial species (i.e., *Bacteroides fragilis*) related to the response [99].

Afterwards, three research teams confirmed these data in humans, reporting the unexpected role of specific members of the gut microbiota as a predictor of response to immunotherapy in a distinctive series of epithelial tumors (NSCLC, renal cell carcinoma, and urothelial carcinoma) and melanoma patients [100,101,102]. Unfortunately, the bacteria genera or species accompanied with the responder phenotype can only partially be matched among these studies [103].

Moreover, the phenotype of responders or nonresponders can be transferred by performing a fecal microbiota transplantation procedure, namely conventionalizing germ-free or antibiotic-pretreated mice with the feces of responder or nonresponder patients [100,101,102]. Similarly, an oral supplementation with specific bacteria (i.e., *Akkermansia muciniphila*) can restore the phenotype of responders in avatar mice obtained from non-responder patients [100].

It is interesting that bacteria involved in the response to checkpoint inhibitors resemble those eating mucin (e.g., *B. longum* or *A. muciniphila*). Theoretically, mucus-eating bacteria could expose a part of the epithelium to themselves or other bacteria, or their antigens [104], thus triggering a proinflammatory response also at a distance.

Zitvogel L. et al. [19] hypothesized different plausible mechanisms of immunostimulation by intestinal bacteria including cross-reactions between microbial and tumor antigens, stimulation of pattern-recognition receptors (PRRs), and production of bacterial metabolites that might exert systemic modulatory effects.

In light of the recent findings of the microbiota as a significant modulator of response to immune checkpoint blockers, it will be interesting to explore the impact of our gut ecosystem on the new concept T-cell-based immunotherapies. Recently, some teams are publishing pioneering works in this field. The gut microbiome and antibiotic therapy appear to impact on the response to adoptive cell therapies in murine models [105,106]. Preliminary data on hematological and solid tumor case series seems to validate this data [107].

Collectively, these and further findings could be deeply significant in order to define plausible combination therapies with microbiota-modulating drugs/foods and the optimal timeline of treatments, given that patients who access this therapy are currently highly pretreated with chemotherapies or other “microbiota-disrupting” therapies. In light of the huge impact of the microbiota on immune system functions and on systemic immune balance, the modulations of its composition or the use of bacterial bioactive compounds, once identified, might gain greater prominence as underpinning therapy to boost the efficacy, or to curtail the toxicities of already available and future immunotherapies [19].

Plausible strategies to fine-tune the microbiota encompass dietary refinement, avoiding improper use of antibiotics, fecal microbial transplantations, and the administrations of prebiotics/probiotics [19,108].

Evidence in mice and human revealed the impact of diet in modulating our microbiome. Intestinal enterotypes are profoundly shaped by long-term diet habits. A prevalence of *Bacteroides* genus and *Prevotella* genus has been associated with an animal protein/fat-based diet and with a plant carbohydrates-based diet, respectively [109]. Furthermore, modifications induced by diet variation occur in a short amount of time [110]. This could impact in modulating the amount of “good” or “bad” bacterial species.

Along these lines, variations in fiber intake can affect the production of short-chain fatty acids (SCFA) and the proportion of potentially beneficial species such as *Faecalibacterium prausnitzii* and *Roseburia spp.* [111]. In particular, SCFA can directly impact on systemic immune regulation through G protein–coupled receptor 43 (GPR43) interaction [112]. Analogously, ω-3 fatty acids can modulate inflammation and insulin sensitivity, interacting with the G protein-coupled receptor 120 (GPR120) [113,114]. These polyunsaturated fatty acids seem also able to induce a transitory increase of some SCFA-producer bacterial genera and to potentially act in restoring eubiosis [115,116].

In view of the above, other modifications of dietary compositions in terms of micro/macronutrient may directly, processed by microbiota or via secondary bacterial/hepatic metabolites, modulate the immune functions and the response to malignant or infectious diseases.

Furthermore, the potential beneficial or noxious effect of nutrients or modern diet habits and subsequently their potential in favorably or detrimentally reshaping the gut compositions will require a closer focus in view of the conceivable implications [117,118].

Preliminary data suggest how lifestyle habits, more specifically diet fiber intake, could impact in terms of odds of response to anti-PD-1 treatment [119].

A further approach could consist of administrating probiotics before, during, or after potentially “microbiota-disrupting” or “microbiota-modulated” treatments. Many trials are currently exploring the effects of these approaches in limiting treatments toxicities, modifying the intratumoral immune response and even impacting on survival outcomes [108].

Focusing on antibiotics, a recent retrospective analysis confirmed how antibiotic treatment prior to immunotherapy (but not concurrently) negatively impacts in terms of overall survival and response rate in cancer patients treated with anti-PD-1/PD-L1 checkpoint inhibitors [120]. Clearly, the infections (respiratory infections were the most common site of infection in the previous series) themselves can exert a negative impact on the outcome of patients (especially in terms of overall survival), but the effect on tumoral response and the great difference reported in terms of survival justify a greater effort to mechanistically understand the reasons of these evidences.

Finally, fecal microbiota transplantation (FMT), which has achieved promising results in the treatment of *Clostridium difficile* infections or refractory IBDs, is going to be evaluated to obtain a recovery of the microbial ecosystem after disrupting treatment such as intensive chemotherapy or allogeneic hematopoietic cell transplantation (NCT03678493), to treat patients with refractory and acute graft versus host disease (NCT03549676, NCT03492502) or to foster the response to immunotherapies in previously “nonresponders” patients (heterologous fecal transplantation from “good-responders”) (NCT03353402) [121,122,123].

## Abbreviations

cfu	colony-forming unit
Tregs	Regulatory T Cells
GALT	Gut-associated lymphoid tissue
CTLA-4	CTLA-4 (Cytotoxic T-Lymphocyte Antigen 4)
PD-1	Programmed cell death protein-1
PD-L1	Programmed cell death-ligand 1
mAbs	Monoclonal antibodies
SFB	Segmented filamentous bacteria
PRRs	Pattern-recognition receptors
TLR4	Toll-like receptor 4
IBD	Inflammatory bowel disease
CAR T	Chimeric antigen receptors T-cell
TCR	T-cell receptor engineered T-cell
SCFA	short-chain fatty acids
FDA	food and drug administration
